# Complex Decongestive Physiotherapy Treats Skin Changes like Hyperkeratosis Caused by Lymphedema

**DOI:** 10.1155/2012/416421

**Published:** 2012-07-01

**Authors:** Hande Kaba, Yesim Bakar, Özlem Çinar Özdemir, Seda Sertel

**Affiliations:** School of Physical Therapy and Rehabilitation, Abant Izzet Baysal University, 14280 Bolu, Turkey

## Abstract

Lymphedema is a chronic, progressive, and often debilitating condition. Primary lymphedema is a lymphatic malformation developing during the later stage of lymph angiogenesis. Secondary lymphedema is the result of obstruction or disruption of the lymphatic system, which can occur as a consequence of tumors, surgery, trauma, infection, inflammation, and radiation therapy. Here, we report a 64-year-old woman presenting with hyperkeratosis, a lymphedema due to metastatic uterus carcinoma. In this paper, we present the effects of complex decongestive physiotherapy on lymphedema and hyperkeratosis.

## 1. Introduction

Lymphedema is a complication, commonly observed in cancer patients. The protein-rich interstitial fluid of lymphedema leads to inflammation and an accumulation of fibroblasts, adiposities, and keratinocytes that transform the initially soft swollen tissue into a hard fibrotic tissue with stiff, thickened skin [1]. At the beginning, the edema does not make any godet. However, thickening is observed in epidermis layer. With disease progression, fibrotic tissue occurs and becomes more stringent [2]. Skin changes especially are observed in chronic lymph edema patients. Specially, if the stasis condition continues for a very long period, skin changes can be observed [3]. These skin changes can be fibrosis, varicose hyperkeratosis, or hyperkeratosis which has high risk of becoming infected [4]. 

Complex decongestive physiotherapy (CDP) is used as a treatment method for lymphedema. This technique includes four parts: (a) manual lymph drainage (MLD), (b) compression bandage, (c) skin care, and (d) remedial exercises. MLD applied in an isolated form is absolutely inadequate. In the drainage area bordering the lymphostatic region, mild strokes are applied. The aim is to stimulate lymphangiomotoric activity. Inside the lymphedematous region itself, the strokes are applied with more pressure to limber up indurate tissues. Compression bandages are constructed in the following manner: for skin protection, one pulls on a cotton sleeve. One inserts upholstering materials with either a smooth surface or a rugged surface followed by textile elastic compression bandages. By using remedial exercises, one activates the muscle and the joint pumps. The aim of skin care is to prevent mycotic and bacterial infections. Skin care starts with hygienic measures. If necessary, disinfective agents are applied; eventually, antimycotics and/or antiallergics are used. In addition to CDP, other methods of physiotherapy often are applied to mobilize joints, to improve the function of the muscle and joint pumps, to rebuild muscles, or to alleviate pains [5, 6].

## 2. Case

**Figure 1:**
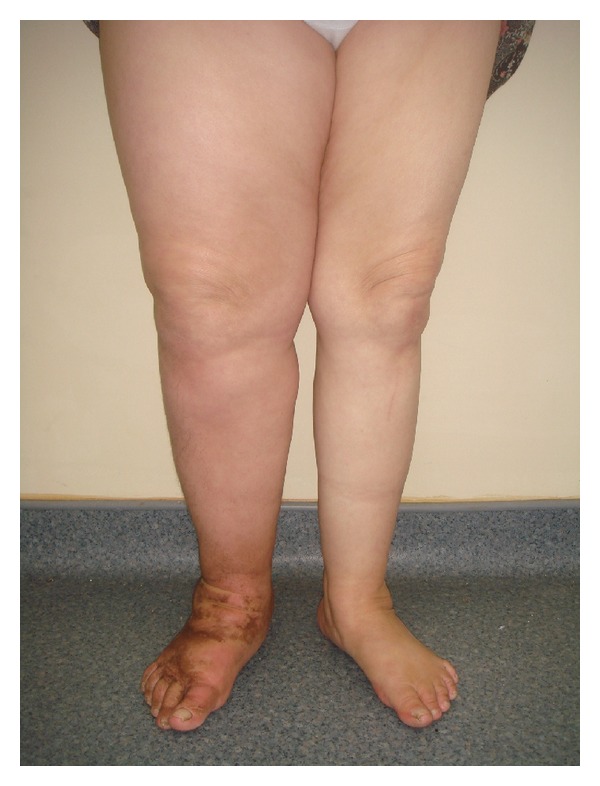


In 2009, when the patient was 62 years old, the mass which size was 19 millimeters was determined on the wall of posterior uterus for using abdominal ultrasonography. The patient has been operated (hysterectomy, bilateral salpingo-oophorectomy, and bilateral pelvic and para-aortic lymph node dissection due to suspicion of metastatic uterus carcinoma). According to the results of a biopsy, metastatic uterus carcinoma was determined for the patient. A 64-year-old woman presented to our department for pain and swelling on her right leg in October 2011. Approximately 1 year after surgery, the patient's right leg has started to swell. It was observed that there was thickening and brown callus on her foot (Figure 1). The patient said to have pain on her whole right back leg. 

The amount of patient's right leg edema was evaluated with Leg Q Meter. Balance disorders caused by edema were measured to use Biodex Balance System SD. Balance was evaluated in two parts, static and dynamic. In this balance assessment, postural stability, fall risk, and sensory integration of balance were observed. Beck depression scale was used to assess psychological status, and Nottingham Health Profile was applied to assess quality of life.

CDP which includes MLD, skin care, compression bandage, and exercises was planned for patients for four weeks. The patient was treated 5 days a week during the treatment period. After the treatment, all of the assessment was repeated again. Only edema evaluation was repeated after 10 days and after the treatment.

## 3. Results

According to the Leg Q Meter assessment, 7 cm slimming of the most swollen part of leg was observed (Table 1).

Stability index of fall risk test did not change both before treatment and after treatment assessment. It was measured as 1.5 for both assessments (Tables 2 and 3).

Beck depression score of the patient was measured as 15 points at the first assessment and 22 points at the second assessment. Although patient respond well to treatment, depression scale score worsened. Although, it was observed that there was improvement of physical status, there was no gain of psychological status. It was determined that the patient had problems about family life and economic status, and these problems affected the patient.

Although improvement of pain was observed, deterioration was detected in other areas (Table 4). It was detected that the patient was affected by reduction of her devotion to life, and problems of her emotional status.

**Figure 2:**
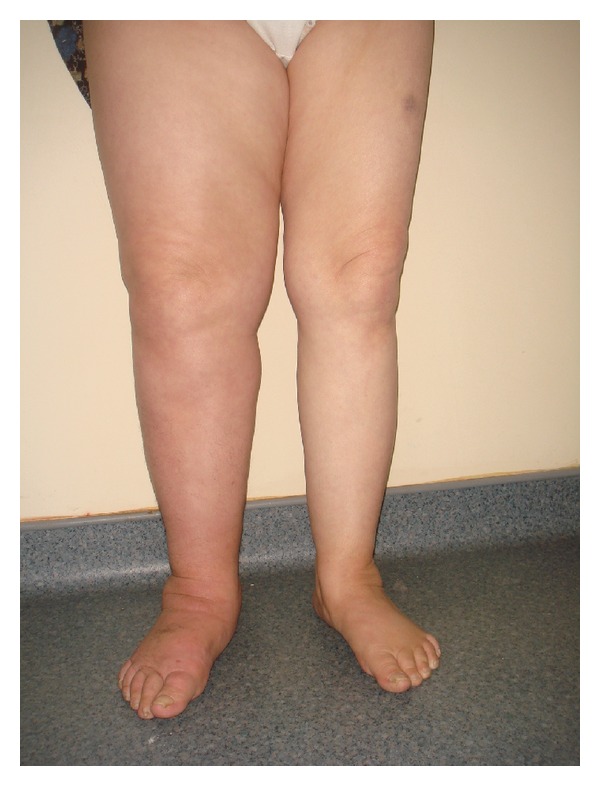


 After treatment, skin began to return to normal color, and after four weeks the brown callus almost disappeared (Figure 2).

## 4. Discussion 

The skin lymphatic system forms a vascular network that drains interstitial fluid from dermis and subcutis and returns it to the blood. Lymphatic vessels are also an important pathway for immune cell trafficking and antigen delivery. Lymphedema is an abnormal swelling condition that may affect one or many body regions. The swelling develops because the lymphatic vessels or nodes have been damaged or were formed incorrectly. Impairment of lymphatic drainage due to (congenital) abnormal vessel development, damaged lymphatic vessels from infection, trauma, surgery, or radiation, and persistent inflammation or obstruction due metastases or parasite infestation cause stagnation of proteins and associated water in the interstitium, which leads to lymphedema [1, 7].

Lymphedema is associated with feelings of discomfort and heaviness, functional limitation, disfigurement, psychological distress, and an elevated risk of recurrent infection. It has a negative effect on quality of life [8, 9]. Although pain and feeling of heaviness decreased, physical activity level worsened in our case. It was determined that emotional status of the patient, problems in family life, economic disadvantage, and environmental factors affected the patient.

Complex physical therapy, also called complex decongestive Physiotherapy, is a treatment regimen that includes meticulous skin hygiene, MLD, bandaging, exercises, and supportive garments. This therapy is carried out in two phases; in the first phase (treatment), the aim is to mobilize the lymph accumulated, reduce the fibrous tissue, and improve the health of the skin using mainly daily MLD during a variable period of time. In addition, patients receive instructions regarding skin care, prophylactic measures, and the use of multilayer bandages. In the second phase (maintenance), compression bandaging, regular physical exercise, and weight control are used [10].

In our study, after treatment with CDP, the skin of the patient gained a healthier appearance. Filling of liquid was blocked with compression bandage; the exercises within bandage increased pumping, venous, and lymphatic circulation. When whole treatment was applied, lymphedema was reduced, and the color of skin turned to normal. It was observed that nearly all of the hyperkeratosis disappeared.

## 5. Conclusion 

Complex decongestive physiotherapy which is used as a treatment for about 30 years is effective on skin changes caused by lymphedema and to reduce edema.

## Figures and Tables

**Table 1:** Leg Q Meter assessment.

	Before treatment		In the middle of assessment (10th session)	After treatment
	Left	Right	Right	Right
Metatarsophalangeal joint	19,6 cm	20,6 cm	20,9 cm	20,3
Ankle	20,8 cm	23 cm	22 cm	21
Achilles tendon	21,6 cm	23,7 cm	21,5 cm	21,5
Gastrosoleus muscle (most swollen region)	32 cm	37,5 cm	33,5 cm	30,4
Head of fibula	29,3 cm	38,1 cm	33 cm	29,8
Middle of knee	33,8 cm	40 cm	38 cm	33,8
Thigh	43,8 cm	54 cm	54,6 cm	53,4
Thigh (most swollen region)	51,6 cm	64,2 cm	64 cm	57

**Table 2:** Biodex balance system SD static postural stability and level 8 assessment.

	Static		Platform level 8 dynamic	
	Before treatment	After treatment	Before treatment	After treatment
Overall	0,8	0,4	1,1	1,4
Anterior/posterior index	0,7	0,4	1,0	1,3
Medial lateral index	0,2	0,2	0,4	0,3

**Table 3:** Biodex balance system sensory integration of balance assessment.

Condition	Sway index	
	Before treatment	After treatment
Eyes open firm surface	0,45	0,66
Eyes closed firm surface	0,80	0,73
Eyes open foam surface	1,38	1,02
Eyes closed foam surface	3,14	2,83

**Table 4:** Nottingham Health Profile.

	Before treatment	After treatment
Energy level	76	100
Pain	100	69,77
Emotional reaction	100	100
Social isolation	63,9	100
Sleeping	100	100
Physical activity	31,29	43,9
Total score	471,19	513,67
